# Integrative analysis of metabolome and gut microbiota in Patients with pancreatic ductal adenocarcinoma

**DOI:** 10.7150/jca.52943

**Published:** 2022-03-06

**Authors:** Xiaodong Guo, Zhengjun Hu, Shu Rong, Guoqun Xie, Gang Nie, Xuan Liu, Gang Jin

**Affiliations:** 1 Department of Oncology, Yueyang Hospital of Integrated Traditional Chinese and Western Medicine, Shanghai University of Traditional Chinese Medicine, ShangHai 200437, China.; 2 Department of Hepatobiliary Pancreatic Surgery, Changhai Hospital Affiliated to Navy Medical University, 168 Changhai Road, Shanghai 200433, China.; 3 Department of Nephrology, Shanghai General Hospital, Shanghai Jiao Tong University School of Medicine, Shanghai 200080, China.; 4 Institute of Interdisciplinary Integrative Biomedical Research, Shanghai University of Traditional Chinese Medicine, ShangHai, 201203, China.

**Keywords:** Pancreatic ductal adenocarcinoma, metabolomics, gut microbiota, biomarkers, 16s rDNA

## Abstract

Pancreatic ductal adenocarcinoma (PDAC) is a highly malignant tumour with a poor prognosis and a high mortality rate. It is of great significance to explore sensitive or specific biomarkers for early diagnosis and prognosis. We first examined the metabolome and gut microbiota of resectable and unresectable PDAC patients to comprehensively investigate the characteristics of PDAC at different stages of progression. At the genus level, we found that the relative abundances of Alistipes, Anaerostipes, Faecalibacterium and Parvimonas were reduced in unresectable PDAC patients, whereas Pseudonocardia, Cloacibacterium, Mucispirillum, and Anaerotruncus were increased. Metabolomics analysis showed that the main changed metabolites were amino acids, carnitine derivatives, lipids and fatty acids. ROC analysis showed that Oleic acid, Linoleic acid, Palmitic acid, Linoelaidyl carnitine, 2-Octenedioic acid, 3R, 7R-1,3,7-Octanetriol, LysoPE (P-16:0/0:0) and 3-Hydroxyanthranilic acid had high AUC values (>0.9). Function and network analyses showed that these altered metabolites correlated with NF-kappa B signalling, the FXR/RXR pathway, mitochondrial dysfunction, mTOR signalling and IL-6 signalling. In particular, the abundance of Palmitic acid, Oleic acid, Linoelaidyl carnitine and 2-Octenedioic acid positively correlated with *g_Anaerostipes, g_Alistipes, s_indistinctus, s_catus and s_formicigenerans* but negatively correlated with *g_Cloacibacterium, s_reuteri and s_hathewayi.* Meanwhile,We also found that *s_catus, s_ formicigenerans, s_ hathewayi, g_ Alistipes, g_ Anaerostipes,* PE (22:6 (4Z, 7z, 10z, 13z, 16Z, 19Z)/p-18:1 (11z)), (3R, 7R) - 1,3,7-octanetriol and linoelaidyl carnitine were positively correlated with the survival time of patients.These findings may be helpful for the differentiation of resectable and unresectable PDAC based on changes in intestinal flora and metabolites at different stages of PDAC. This study also provides a strategy for preventing the deterioration of PDAC by regulating the gut microbiota and metabolism.

## Introduction

Pancreatic duct adenocarcinoma (PDAC) is one of the most malignant tumours. According to data from the American Cancer Society in 2020, the death rate of pancreatic cancer in the United States ranks third among malignant tumours and second among digestive system tumours^1^. Statistics from the China National Cancer Center also showed that the incidence of PDAC was the ninth highest among malignant tumours in China, and the mortality rate was sixth, with a very poor prognosis^2^. However, in contrast to the high mortality rate, pancreatic cancer has a low survival rate, With a 5-year survival rate of less than 10%^1^^1^. The main reason for this is that the rate of radical operation is too low. At present, less than 20% of patients with primary pancreatic cancer can be operated on^3^. It has been reported that the median survival time of patients with resectable pancreatic cancer after adjuvant chemotherapy is between 20.1 and 28.0 months, while the median survival time (MST) of patients with locally advanced and metastatic pancreatic cancer after treatment with folfirinox and gemcitabine plus nab-paclitaxel is only 8.5 to 11.1 months^4^,^5^. There was a significant difference in survival benefit between patients who could and could not undergo operation. Thus, in recent years, clinical studies have been trying to improve the resectability of locally advanced and partially metastatic pancreatic cancer through new treatment. However, patients who were originally classified as borderline resectable pancreatic cancer have been treated with neoadjuvant chemotherapy to improve the R0 resection. Therefore, the early detection and diagnosis of pancreatic cancer is very important to improve the radical operation rate.

Due to the anatomical location of the pancreas, the onset of pancreatic cancer is more insidious, and the clinical symptoms are not obvious and lack specificity. Therefore, most pancreatic cancers are diagnosed at a late stage. CA199 is the most effective and clinically valuable biomarker for the early detection of pancreatic cancer recommended by the NCCN guidelines, with a sensitivity of 79% to 81% and specificity of 80% to 90% for symptomatic patients^6^. In recent years, with the development of genomics, proteomics and metabonomics, increasing biomarkers of pancreatic cancer have been found. Metabonomics may be more sensitive in this regard^7^. Significant differences have been found between the blood and urine metabolites of pancreatic cancer patients and healthy people^8^,^9^.

Changes in microbial genera have also been related to pancreatic cancer. It has been found that *Fusobacterium* increases in pancreatic cancer tissue, and there is a higher mortality rate and shorter survival period in cases positive for *Fusobacterium* 10. At the same time, the average relative abundances of *Fusobacterium*,* Porphyromonas*,* idocharacter*,* Capnocytophaga*,* Prevotella, Selenomonas* and *Gemella* in pancreatic cancer patients were higher than those in healthy people 11. In fact, changes in the metabolites and flora *in vivo* are highly correlated, especially in cancer patients, which jointly affect the progression of cancer. However, research has not yet simultaneously characterized the different periods of pancreatic cancer from these two aspects.

In this study, we first examined the metabolome and gut microbiota of resectable PDAC and unresectable PDAC patients and and discussed the correlation between specific gut microbiota, specific metabolome and the survival time of patients.Our findings depict a novel picture of the gut microbiota in PDAC with respect to metabolites, aiming to find markers and possible mechanism of disease progression of PDAC.

## Methods

### Patients and samples

Resectable (n=36) and unresectable PDAC (n=36) patients were recruited from the the department of Hepatobiliary Pancreatic Surgery, Changhai Hospital Affiliated with Navy Medical University and Yueyang Hospital of Traditional Chinese and Western Medicine Affiliated to Shanghai University of Traditional Chinese Medicine. All the patients recruited for the study have provided written informed consent for use of patient samples and data. All patients were confirmed as pancreatic ductal adenocarcinoma by pathological examination. The histopathological results of resectable PDAC patients were obtained after operation. Unresectable PDAC patients obtained pathological examination by endoscopic ultrasound-guided fine needle aspiration (EUS-FNA). All patients were examined by thin-section computed tomography and magnetic resonance imaging of the abdomen before admission, and blood biochemical parameters and tumour markers were detected. All patients were further subjected to multidisciplinary discussions to plan the optimal treatment strategy. The inclusion criteria were as follows: age ranged from 50-80 years old; primary-care patients without any treatment; had quit smoking and drinking alcohol for more than half a year; did not take antibiotics in the past month. The exclusion criteria were as follows: other basic diseases, such as patients with a history of diabetes were excluded, hypertension, arthritis, heart disease, chronic hepatitis, gallstone, Alzheimer's disease; unclear medication history; those who could not fully cooperate with baseline data collection. All patients were diagnosed with pancreatic cancer for the first time. Blood was gained from all the patients though vein with fasting abdomen in the morning.

### Definition of resectable PDAC and unresectable PDAC

The diagnostic criteria of resectable PDAC refer to the NCCN guidelines for pancreatic adenocarcinoma^12^: the solid tumour did not contact the coeliac trunk artery, superior mesenteric artery, hepatic artery, superior mesenteric vein or portal vein; or it contacted it but did not exceed 180 degrees, and the vein contoured regularly; there was no distant metastasis. Unresectable PDAC included patients with local advanced and metastatic pancreatic cancer. Locally advanced stage was defined as solid tumour contacting the coeliac trunk artery or superior mesenteric artery by more than 180 degrees; solid tumour contacting the aorta ventral and the coeliac trunk artery; tumour invasion or embolism leading to superior mesenteric vein and portal vein, which could not be resected or reconstructed. Metastatic pancreatic cancer refers to distant metastasis.

### DNA Extraction and Sequencing

DNA was extracted from frozen faecal matter (200 mg) with a PowerFecal DNA Isolation Kit (cat: 12830-50, QIAGEN-MOBIO) according to the HMP (Human Microbiome Project) guidelines. PCR amplification of 16S rDNA involved thirty-five PCR cycles (95 ℃, 35 s, 55 ℃ for 35 s and 72 ℃ for 1 min). The forward primer is 5'-AGAGTTTGATCCTGGCTCAG-3', and the reverse primer is 5'-AGAGTTTGATCCTGGCTCAG-3'. The successful amplification product was verified by agarose gel electrophoresis, and DNA libraries were constructed according to the manufacturer's instructions of KAPA Library Quantification kits and LabChip GX HT DNA High Sensitivity Kit (Waltham, MA, USA). Sequencing was performed on the Illumina MiSeq platform.

### Metabolic Sample Preparation and Analysis

In total, 100 µL of serum sample was added to 300 µL methanol and then vortex-mixed for 1 min. The mixture was then centrifuged at 12,000 rpm for 15 min at 4 ℃, and the supernatant was transferred into a vial. The LCMS detection was performed on an Agilent 1290 (UPLC) system equipped with an Agilent 6545 QTOF mass spectrometer (MS) (Agilent, Waltham, MA, USA) in the positive ion mode and negative mode. Waters T-3 column (100 mm x 2.1 mm; 1.8-µm particle size) was used for the separation of metabolites with a column temperature of 40 ℃ and an injection volume of 2 µL. The flow rate was 0.3 mL/min, and the mobile phases consisted of 0.1% formic acid (v/v) in ultra-pure water (mobile phase A) and 0.1% formic acid (v/v) in acetonitrile (mobile phase B). The gradient elution used was: 0-2 min, 2%-2% B; 2-10 min, 2-95% B; 10-12 min, 95% B; 12-15 min, 95-2% B. Data acquisition was performed on an Agilent Mass spectrometer with the following mass parameters: the flow rate of drying gas N_2_ was 11 L/min, the source temperature was set at 360 ℃, the pressure of the nebulizer was 40 psi, the capillary voltage was 4100 V (positive ionization) and 4000 V (negative ionization mode), and the mass scan range was from 60 to 1500.

### Metabolic Data Analysis

The raw data were converted to mzdata with MassHunter Workstation Quantitative Analysis B.08. R software 3.6.3, and the XCMS package was used for peak extraction, peak alignment, peak matching, and finally to generate an ion list containing the retention time and mass to charge ratio (m/z) values. After normalization, the table list of ions was imported into SIMCA-P 13.0 and UV-scaled for multivariate statistical analysis. First, Principal Component Analysis (PCA), an unsupervised method of analysis, was used to show the differences in metabolic status between the two groups. Then, Partial least squares discriminant analysis (PLS-DA), a supervised analysis method, was used to screen the differentially expressed metabolites. Finally, the different metabolites were selected with *p* < 0.05, VIP >1 and fold change (FC > 1.2). The differential metabolites were identified by comparing exact masses with metabolite databases, including HMDB and METLIN. Then, MS/MS spectra were used to confirm the metabolites structure. Finally, the retention time MS/MS spectra were compared with standard compounds obtained from Sigma-Aldrich to confirm the metabolite structures, followed by metabolic pathway or function analysis using the KEGG and HMDB databases. Correlation analysis of different metabolites and different bacteria used Pearson correlation analysis based on R software (http://www.r-project.org). The Pearson correlation coefficients were generated and declared significant at p < 0.05.

### 16s rDNA data analysis

The original data were processed by dejoining and low-quality filtering, and chimeric sequences were removed. After clean data were obtained, cluster analysis was carried out with QIIME (version 1.9.1), and Operational Taxonomic Units (OTUs) were acquired. The representative sequences of each OUT were annotated and aligned to obtain the corresponding species information and species abundance distributions. At the same time, the abundance, alpha diversity, Venn map and petal map of OTUs were analysed to obtain species richness and evenness information using the QIIME software package.

## Results

### Clinical Characteristics of the included patients

In all, 72 patients were finally included for analysis, including 36 resectable PDAC patients and 36 unresectable PDAC patients. The two groups showed no significant differences in age, gender, or body mass index (Table 1). The levels of CA199, CA125, and CEA were higher in unresectable PDAC patients than resectable PDAC group. In the resectable group, the stages of cancer were mainly stage I/II (30/6), and the tumour location was at the head of the pancreas (24) or body/tail of the pancreas (12) with no metastasis. In the unresectable group, the stages of cancer were mainly stage III/IV (9/27), and the tumour location was at the head of the pancreas (14) or body/tail of pancreas (22), with observed metastasis of the lung (4), liver (13), abdominal lymph nodes (6) and adrenal gland (6).

### Differently expressed metabolites analysis

**Figure 1:**
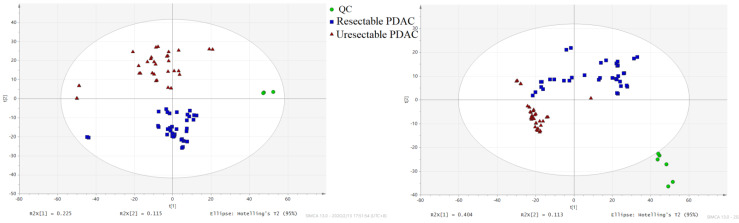
PCA score plot. (■) Resectable PDAC group; (▲) unresectable PDAC; (●) QC sample.

**Figure 2:**
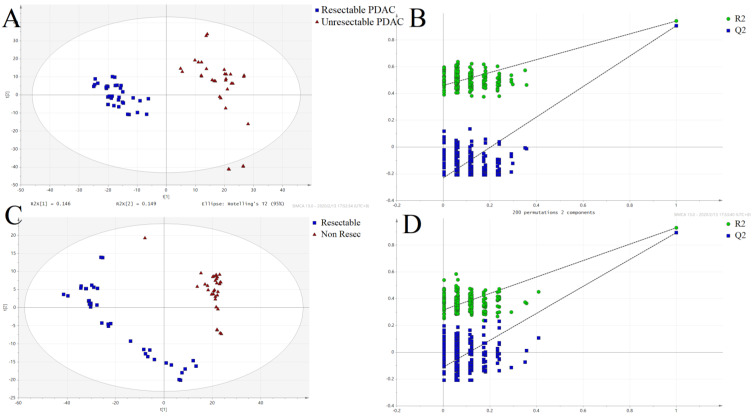
Partial least squares-discriminant analysis (PLS-DA) score plots and permutation test for the model discriminating serum samples from resectable and unresectable PDAC patients. **(A)** POS-PLS-DA score plot; **(B)** POS-permutation test for the model of PLS-DA; **(C)** NEG-PLS-DA score plot; **(D)** NEG-permutation test for the model of PLS-DA: (■) resectable PDAC group, (▲) unresectable PDAC group.

**Figure 3:**
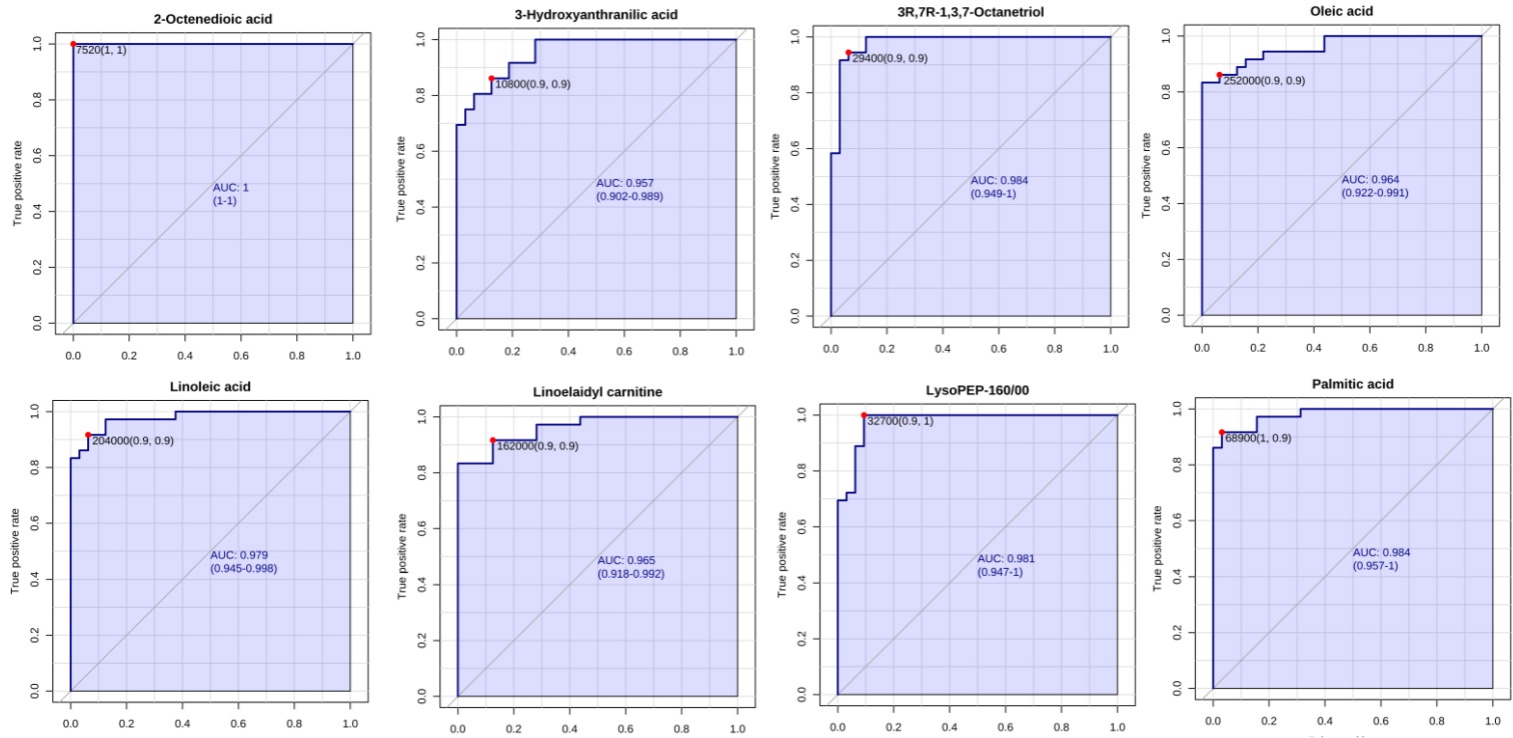
ROC curve analysis of potential serum biomarker levels for differentiating unresectable from resectable PDAC.

**Figure 4:**
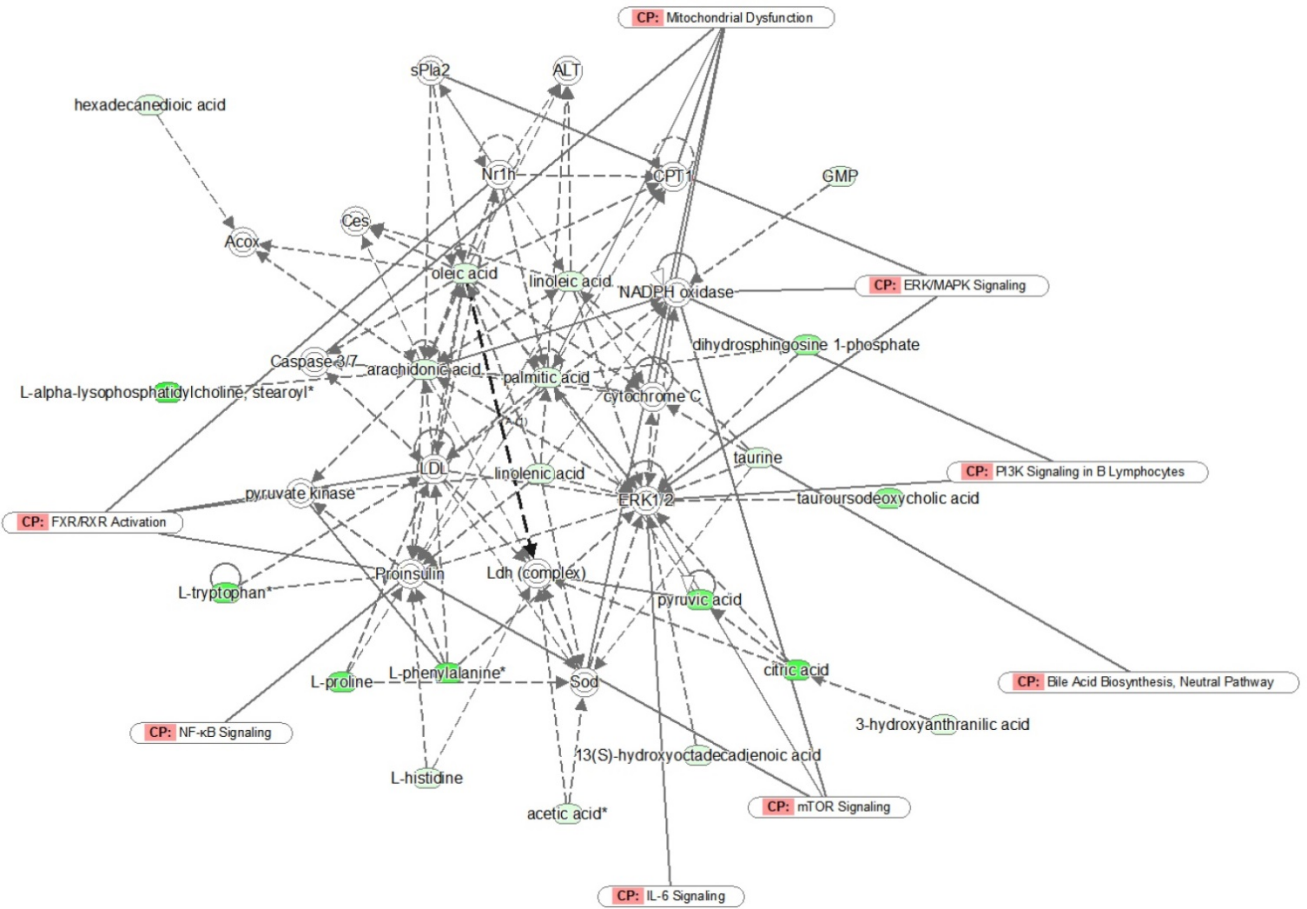
Network analysis of the identified metabolites by IPA. Green symbols represent downregulated metabolites in unresectable PDAC patients; CP represents canonical pathways that are related to the identified specific metabolites. Solid lines represent a direct relationship between molecules, while dotted lines represent indirect relationships.

The PCA score showed different separation trends of the two groups (Fig. 1), indicating that the metabolism of the two groups was completely different. PLSDA analysis (Fig. 2) was then used to screen the different metabolites, and we identified 149 differentially expressed metabolites (VIP > 1, P < 0.05, FC > 1.2; Table S1), which were mainly divided into carnitine, long-chain fatty acids, phospholipids and amino acids. ROC analysis was carried out for these metabolites, showing that 11 of these metabolites had high AUC values (Table 2.), including Oleic acid, Linoleic acid, Palmitic acid, Linoelaidyl carnitine, 2-Octenedioic acid, 3R,7R-1,3,7-Octanetriol, LysoPE (P-16:0/0:0) and 3-Hydroxyanthranilic acid (AUC > 0.9), with high sensitivity and specificity (Fig. 3). Function and network analyses showed that these altered metabolites were correlated with the NF-κB signalling pathway, FXR/RXR activation pathway, mitochondrial dysfunction, mTOR signalling and IL-6 signalling (Fig. 4.).

### Diversity and Relative Abundance of Taxa

**Figure 5:**
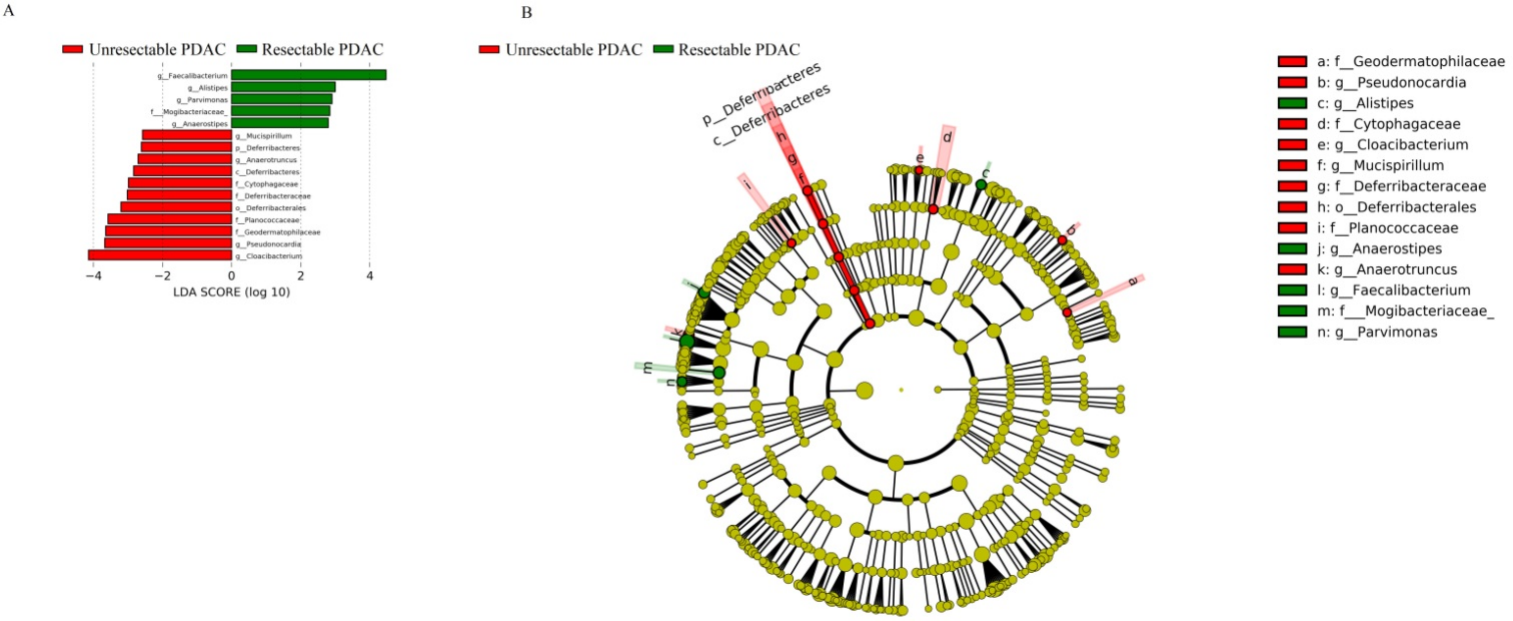
LEfSE Plot. A, LDA score. The histogram of LDA value distribution shows the species with significant differences in abundance between groups, and the length of the histogram represents the impact of different species. The red column represents unresectable PDAC patients, and green represents resectable PDAC patients; B, Evolutionary branch map. The circle radiating from the inside to outside represents the classification level from phylum to genus (or species). Each small circle at different classification levels represents a classification at that level, and the diameter of the small circle is proportional to the relative abundance. Species without significant differences are uniformly coloured yellow. The red node represents the microbial group that plays an important role in the red group, and the green node represents the microbial group that plays an important role in the green group.

The two groups did not show differences in the Chao index, a measure of within-sample (α) phylogenetic diversity (Figure S1). The top 15 species by average abundance are shown in Figure S2, and the differential bacteria, at the level of order, family, genus and species, are shown in Table 3. At the genus level, the relative abundances of *Alistipes*,* Anaerostipes*,* Faecalibacterium*, and* Parvimonas* were reduced in unresectable PDAC patients (p < 0.05), and *Pseudonocardia*,* Cloacibacterium*,* Mucispirillum*, and* Anaerotruncus* were increased (Fig 5).

### Correlation between the gut microbiome and metabolites

**Figure 6:**
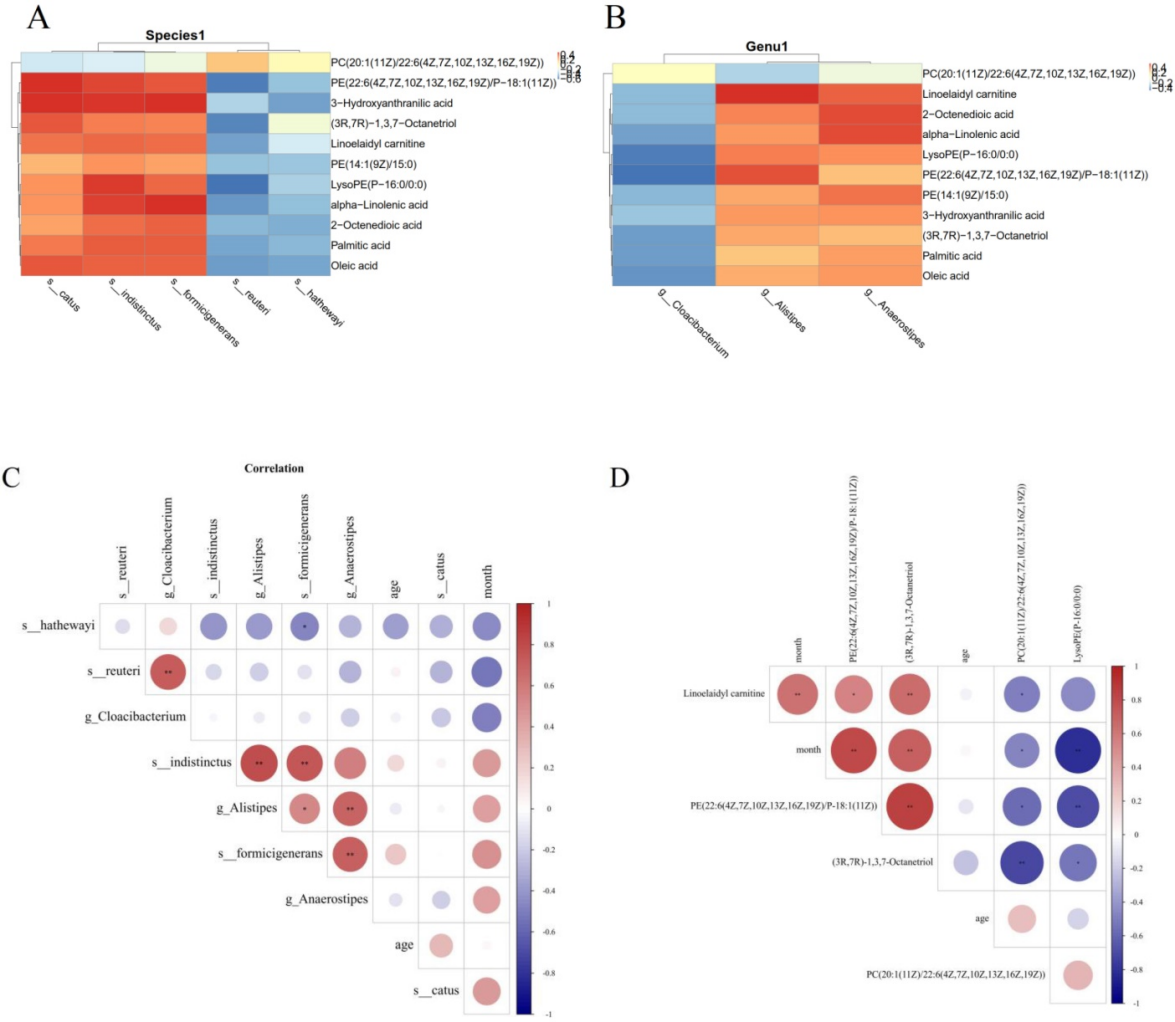
Correlation analysis of metabolites and different species; (A) and genera (B). (C) Correlation analysis of survival time of PDAC patients and intestinal flora;(D)Correlation analysis of survival time of PDAC patients and metabolites.

The Pearson's correlation coefficient was used to explore the correlation between the changed gut microbiome and metabolite perturbations (Fig 6). Clear correlations could be identified between the perturbed gut microbiome and altered metabolite profiles (p < 0.05). Figure 6 shows that several typical gut microflora were highly correlated with specific metabolites. *s_indistinctus*,* s_catus* and *s_formicigenerans* showed positive correlations with Palmitic acid, Oleic acid, Linoelaidyl carnitine and 2-Octenedioic acid, while *s_reuteri* and* s_hathewayi* showed negative correlations with Palmitic acid, Oleic acid, Linoelaidyl carnitine and 2-Octenedioic acid (p < 0.05) (Fig 6A). Palmitic acid, Oleic acid, Linoelaidyl carnitine and 2-Octenedioic acid positively correlated with the *g_Anaerostipes* and* g_Alistipes* but negatively correlated with *g_Cloacibacterium*. Likewise, LysoPE (P-16:0/0:0) negatively correlated with *g_Cloacibacterium*, but positively correlated with *g_Anaerostipes* and* g_Alistipes* (p < 0.05) (Fig 6B). We also found that *s_catus, s_ formicigenerans, s_ hathewayi, g_ Alistipes and g_ Anaerostipes* were positively correlated with the survival time of patients, suggesting that these bacteria were the protective factor. PE (22:6 (4Z, 7z, 10z, 13z, 16Z, 19Z)/p-18:1 (11z)), (3R, 7R) - 1,3,7-octanetriol and linoelaidyl carnitine were positively correlated with patient survival time, PC (20:1 (11z) / 22:6 (4Z, 7z, 10z, 13z, 16Z, 19Z)) and LysoPE (p-16:0/0:0) was negatively correlated with survival time.This is what distinguishes us from other studies. This part of the research results have been added in the article, as shown in Fig 6C, D.

## Discussion

Radical resection is the most effective treatment for pancreatic cancer. However, more than 80% of pancreatic cancer patients do not have the option of operation due to late diagnosis. Therefore, it is of great significance to explore sensitive or specific biomarkers for early diagnosis and prognosis. The development of High-Throughput Sequencing technology and Metabonomics has provided the possibility for this exploration. The occurrence and development of pancreatic cancer are bound to be accompanied by changes in the flora and metabolites in the body. Mouse models have been used to observe early and late pancreatic intraepithelial neoplasia to simulate the malignant progress of PDAC in human disease. It has been found that serum citrate levels increase in late pancreatic intraepithelial neoplasia^13^, and the concentrations of carnitines, such as C5 acyl carnitine and propionyl carnitine, are negatively correlated with the progression of pancreatic cancer 14. Studies have also found some microbes in the tumour tissue related to the survival of unresectable PDAC patients^15^. These findings suggest that the monitoring of metabolites and microorganisms may provide valuable clues for the development and prognosis of pancreatic cancer. It can be concluded that resectable pancreatic cancer and locally advanced and metastatic pancreatic cancer are different stages of disease development, with different survival and prognosis. We first used metabonomics and 16SrDNA to characterize resectable and unresectable PDAC to more comprehensively understand the physiological state of patients in these two stages and to identify screening markers that can be used to judge the disease prognosis. This type of data is very meaningful for the early detection and intervention of PDAC.

In our study, we found that the content of carnitine and fatty acids was significantly reduced in unresectable PDAC. One of the main functions of carnitine is to transport long-chain fatty acids from the cytoplasm through the mitochondrial membrane into the mitochondrial matrix for β oxidation, which produces energy. The enhanced β oxidation of long-chain fatty acids in the mitochondria is an important energy source of PDAC^16^. It has been confirmed that L-carnitine deficiency is an important cause of cancer cachexia and cancer-related fatigue^17^. In our study, compared with the resectable group, the content of carnitine and fatty acids in unresectable PDAC was significantly reduced, suggesting that tumour growth needs more energy and accelerates the β oxidation of fatty acids in unresectable PDAC patients.

Many studies have confirmed that saturated and monounsaturated long-chain fatty acids can promote the progression of pancreatic cancer^18^,^19^, which is related to the accelerated oxidation of long-chain fatty acids to promote energy production in cancer cells^16^. Meanwhile, ѡ-3 polyunsaturated fatty acids can inhibit the proliferation of pancreatic cancer cells^18^,^19^. In our study, we found that the contents of palmitic acid (saturated fatty acid), oleic acid (monounsaturated fatty acid), docosahexaenoic acid and alpha-linolenic acid (ѡ-3 polyunsaturated fatty acid) and other long-chain fatty acids were significantly decreased in patients with unresectable PDAC compared to patients with resectable PDAC, while the abundance of *Alistipes* in the faeces was significantly decreased. Because of the relationship between intestinal microorganisms and fatty acid metabolism, we also found a positive correlation between saturated long-chain fatty acids and the abundance of *Alistipes* in our study, which is consistent with the previous literature^20^. It also suggests that changes in intestinal microorganisms and serum fatty acids can be used to evaluate the progression of pancreatic cancer.

The main components of eukaryotic cell membranes are phospholipids, including phosphatidylserine (PS), phosphatidylcholine (PC), phosphatidylethanolamine (PE), phosphatidylinositol (PI) and phosphatidylic acid, which participate in a variety of biological functions^21^. It also changes in many types of tumours, including pancreatic cancer^22^. We found that the levels of lysophosphatidylcholine (LPC) and phosphatidylcholine in the serum of patients with unresectable PDAC also decreased significantly because the tumour absorbs lipids in the microenvironment to meet the needs of fatty acid consumption and proliferation under the conditions of pancreatic cancer. In addition, PDAC cells use lysophosphatidylcholine support phosphatidylcholine synthesis, which is used to form cell membranes. In addition, LPC is hydrolysed by the secreted autotaxin in the extracellular space to produce lysophosphatidic acid (LPA). LPA can promote the proliferation and metastasis of pancreatic cancer by binding to the LPA receptor coupled with G protein^23^. Interestingly, another study has shown that phosphatidylcholine deficiency may promote the growth of pancreatic cancer and accelerate the progression of pancreatic cancer^24^, which is consistent with our results. The lower levels of lysophosphatidylcholine and phosphatidylcholine in serum may indicate that pancreatic cancer is in a more serious physiological state. The abundance of *Faecalibacterium* was reduced in unresectable PDAC and positively correlated with phosphatidylcholine in our study. *Faecalibacterium* plays an important role in various cancers. It may be helpful for the prevention of breast cancer, as reductions of *Faecalibacterium* promote the development of breast cancer^25^. It was verified that *Faecalibacterium prausnitzii* improved lung cancer disease by upregulating the expression of anti-inflammatory cytokines and downregulating the expression of pro-inflammatory cytokines^26^. Our study indicated that *Faecalibacterium* may be helpful for the prevention of PDAC, and adding *Faecalibacterium* may slow down its progression, while further experiments are needed to verify our conclusion. LysoPE (P-16:0/0:0) was decreased in unresectable PDAC, while *Anaerotruncus* was increased. Interestingly, in colorectal cancers, LysoPE (P-16:0/0:0) has displayed significant relationships with *Anaerotruncus*^27^.

Tryptophan is an essential amino acid that plays an important role in protein biosynthesis and metabolic network regulation. Tryptophan is mainly metabolized through the following pathways: canine urinary ammonia, 5-hydroxytryptamine pathway and bacterial degradation. Most tryptophan (95%) is metabolized into a series of downstream products through the kynurenine pathway^28^. This pathway is involved in inflammation and immune function through the consumption of tryptophan and the immunoregulation of downstream products^29^. Our results showed that the level of tryptophan in unresectable PDAC was significantly lower than that in resectable PDAC. IDO is the rate limiting enzyme responsible for tryptophan degradation. Research has shown that IDO is highly expressed in pancreatic cancer tissues, especially in tissues with lymph node metastasis and low differentiation^30^. As a result, the level of tryptophan in serum is reduced. The above studies showed that the uric acid metabolic pathway consumed by tryptophan increased significantly in pancreatic cancer and was related to its progression and invasiveness. Therefore, the kynurenine pathway is a significant therapeutic target^31^.

In conclusion, this study first examined the metabolic and microbiome differences between resectable and unresectable PDAC patients. and discussed the correlation between specific gut microbiota, specific metabolome and the survival time of patients.These findings may assist in distinguishing resectable and unresectable PDAC patients based on changes of potential biomarkers. The data also provide a strategy for preventing the deterioration of PDAC by regulating the gut microbiota and metabolism.

## Supplementary Material

Supplementary figures and table.

## Figures and Tables

**Table 1:** The clinical characteristics of unresectable and resectable PDAC patients.

	Resectable PDAC	Unresectable PDAC
Age		
median	59	63
Range	(50-72)	(59-80)
Gender		
Male	20	24
Female	16	12
BMI	23.1 ± 1.9	21.3 ± 2.5
Stages		
I/II	30/6	0
III/IV	0	9/27
Tumor location		
Head	24	14
Body/Tail	12	22
Pre-treatment CA199 Median range	226.4 (2.04-1200)	22517.1 (1.2-121248.9)
Pre-treatment CA125 Median range	19.8 (7.8-41)	333.5 (2.45-2788.4)
Pre-treatment CEA Median range	3.6 (1.2-9.1)	46.1 (1.5-470.3)
Metastasis	No	No (9) , lung (4) , liver (13), Abdominal lymph nodes (6), Adrenal gland (6)

**Table 2:** Differential metabolites in unresectable and resectable PDAC patients.

VIP	mz	RT	HMDB_ID	Adduct	Name	Formula	P	LOG2FC(un/res)
2.06	163.1328179	5.70	HMDB0033625	M+H	(3R,7R)-1,3,7-Octanetriol	C8H18O3	4.21E-13	-29.61
1.59	171.0653969	5.38	HMDB0000341	M-H	2-Octenedioic acid	C8H12O4	1.41E-20	-1.31
1.33	207.0405978	5.30	HMDB0000341	M+Cl	2-Octenedioic acid	C8H12O4	1.99E-09	-5.44
1.29	188.0170452	1.62	HMDB0001476	M+Cl	3-Hydroxyanthranilic acid	C7H7NO3	2.55E-13	-1.52
2.04	424.3421819	9.14	HMDB0006461	M+H	Linoelaidyl carnitine	C25H45NO4	4.76E-13	-34.85
1.34	279.2325866	11.10	HMDB0000673	M-H	Linoleic acid	C18H32O2	4.84E-15	-1.54
1.96	438.2982461	9.46	HMDB0011152	M+H	LysoPE(P-16:0/0:0)	C21H44NO6P	1.10E-11	-29.85
1.35	281.2481374	11.66	HMDB0000207	M-H	Oleic acid	C18H34O2	6.56E-15	-1.48
1.33	255.2324846	11.53	HMDB0000220	M-H	Palmitic acid	C16H32O2	8.90E-15	-1.64
1.16	882.5904013	13.15	HMDB0008321	M+Na	PC(20:1(11Z)/22:6(4Z,7Z,10Z,13Z,16Z,19Z))	C50H86NO8P	3.64E-04	-30.40
1.30	682.4040779	5.41	HMDB0008856	M+Cl	PE(14:1(9Z)/15:0)	C34H66NO8P	1.21E-13	-4.01
1.33	646.4261523	11.65	HMDB0008856	M-H	PE(14:1(9Z)/15:0)	C34H66NO8P	1.97E-11	-1.07
2.11	796.5259999	11.09	HMDB0009710	M+Na	PE(22:6(4Z,7Z,10Z,13Z,16Z,19Z)/P-18:1(11Z))	C45H76NO7P	2.37E-14	-35.09
2.02	796.526071	11.65	HMDB0009710	M+Na	PE(22:6(4Z,7Z,10Z,13Z,16Z,19Z)/P-18:1(11Z))	C45H76NO7P	1.09E-12	-36.20
1.23	772.5299073	11.10	HMDB0009710	M-H	PE(22:6(4Z,7Z,10Z,13Z,16Z,19Z)/P-18:1(11Z))	C45H76NO7P	3.39E-08	1.16

FC(un/res) represent the fold change of unresecble PDAC/ Resecble PDAC patients.

**Table 3:** Information of differentially expressed bacteria at the level of order, family, genus and species.

Class	ID	FC(un/re)	p
Family	k_Bacteria;p_Cyanobacteria;c_4C0d-2;o_YS2;f_	11.33	0.04
	k_Bacteria;p_Bacteroidetes;c_Bacteroidia;o_Bacteroidales;f_[Barnesiellaceae]	0.17	0.04
Order	k_Bacteria;p_Cyanobacteria;c_4C0d-2;o_YS2	11.33	0.04
	k_Bacteria;p_TM7;c_TM7-3;o_CW040	3.47	0.05
Species	K_Bacteria;p_Firmicutes;c_Bacilli;o_Lactobacillales;f_Lactobacillaceae;g_Lactobacillus;s_reuteri	7.49	0.01
	k_Bacteria;p_Bacteroidetes;c_Bacteroidia;o_Bacteroidales;f_Rikenellaceae;g_Alistipes;s_indistinctus	0.01	0.01
	k_Bacteria;p_Firmicutes;c_Clostridia;o_Clostridiales;f_Lachnospiraceae;g_Coprococcus;s_catus	0.03	0.03
	k_Bacteria;p_Firmicutes;c_Clostridia;o_Clostridiales;f_Lachnospiraceae;g_Dorea;s_formicigenerans	0.43	0.04
	k_Bacteria;p_Cyanobacteria;c_4C0d-2;o_YS2;f_;g_;s_	0.09	0.04
	k_Bacteria;p_Bacteroidetes;c_Bacteroidia;o_Bacteroidales;f_[Barnesiellaceae];g_;s_	6.00	0.04
	k_Bacteria;p_Firmicutes;c_Clostridia;o_Clostridiales;f_Lachnospiraceae;g_Clostridium;s_hathewayi	16.91	0.04
Genus	k_Bacteria;p_Bacteroidetes;c_Bacteroidia;o_Bacteroidales;f_Rikenellaceae;g_Alistipes	0.27	0.02
	k_Bacteria;p_Bacteroidetes;c_Flavobacteriia;o_Flavobacteriales;f_[Weeksellaceae];g_Cloacibacterium	10^4^	0.03
	k_Bacteria;p_Firmicutes;c_Clostridia;o_Clostridiales;f_Lachnospiraceae;g_Anaerostipes	0.25	0.03

FC(un/res) represent the fold change of unresecble PDAC/ Resecble PDAC patients.
