# Supplementary material for: Integrative analysis of metabolome and gut microbiota in Patients with pancreatic ductal adenocarcinoma
Source: J Cancer. 2022 Mar 6;13(5):1555–64. doi: 10.7150/jca.52943 (PMC8965132; doi:10.7150/jca.52943)

Supplemental Material

Integrative analysis of metabolome and gut microbiota in Patients with pancreatic ductal adenocarcinoma

Xiaodong Guo[1, *, #], Zhengjun Hu[1, #], Shu Rong[3, #], Guoqun Xie[1], Gang Nie[2], Xuan Liu[4], Gang Jin[2, *]

1. Department of Oncology, Yueyang Hospital of Integrated Traditional Chinese and Western Medicine, Shanghai University of Traditional Chinese Medicine，ShangHai 200437, China.

2. Department of Hepatobiliary Pancreatic Surgery, Changhai Hospital Affiliated to Navy Medical University, 168 Changhai Road, Shanghai 200433, China.

3. Department of Nephrology, Shanghai General Hospital, Shanghai Jiao Tong University School of Medicine, Shanghai 200080, China.

4. Institute of Interdisciplinary Integrative Biomedical Research, Shanghai University of Traditional Chinese Medicine, ShangHai, 201203, China.

* Corresponding author: Xiaodong Guo, Department of Oncology, Yueyang Hospital of Integrated Traditional Chinese and Western Medicine, Shanghai University of Traditional Chinese Medicine，ShangHai 200437, China.

E-mail address: gxd_1996@shutcm.edu.cn.

Gang Jin, Department of Hepatobiliary Pancreatic Surgery, Changhai Hospital Affiliated to Navy Medical University, 168 Changhai Road, Shanghai 200433, China.

E-mail address: jingang@sohu.com

# Both authors contributed equally to this work.

Supplemental Material, Table S1. Identified significantly changed metabolites (fold change >1.5 $p < 0.05$ compared to resectable PDAC) in serum samples.

VIP	mz	RT	HMDB_ID	Adduct	Name	P	FC(UN/RES)
1.63	520.3396	8.92	HMDB0010386	M+H	LysoPC(18:2(9Z,12Z)/0:0)	1.44E-07	1.74E-14
1.37	522.3554	9.48	HMDB0002815	M+H	LysoPC(18:1(9Z)/0:0)	2.18E-05	3.51E-14
1.63	518.3218	9.24	HMDB0240262	M+Na	LysoPC(0:0/16:0)	1.15E-07	5.05E-13
1.15	546.3532	10.19	HMDB0010384	M+Na	LysoPC(18:0/0:0)	4.39E-04	9.57E-13
1.49	193.0346	1.09	HMDB0000094	M+H	Citric acid	2.13E-06	1.05E-12
1.11	464.3350	5.68	HMDB0013339	M+Na	3-Hydroxy-11Z-octadecenoylcarnitine	1.31E-03	1.90E-12
1.14	302.3054	8.33	HMDB0000269	M+H	Sphinganine	6.06E-04	2.39E-12
1.07	566.4282	5.81	HMDB0011497	M+H	LysoPE(0:0/24:0)	1.90E-03	2.84E-12
1.83	205.0972	4.01	HMDB0000929	M+H	L-Tryptophan	6.85E-10	3.06E-12
1.11	546.3554	9.26	HMDB0010393	M+H	LysoPC(20:3(5Z,8Z,11Z)/0:0)	6.56E-04	5.08E-12
1.32	494.3242	8.64	HMDB0010383	M+H	LysoPC(16:1(9Z)/0:0)	3.67E-05	7.42E-12
1.22	116.0708	0.79	HMDB0000162	M+H	L-Proline	1.65E-04	1.06E-11
1.19	688.5205	10.30	HMDB0007928	M+H	PC(14:1(9Z)/P-16:0)	2.61E-04	1.07E-11
1.77	480.3362	9.50	HMDB0010407	M+H	LysoPC(P-16:0/0:0)	4.28E-09	1.21E-11
1.38	426.3577	9.59	HMDB0005065	M+H	Oleoylcarnitine	1.55E-05	1.25E-11
1.47	746.5639	10.73	HMDB0007938	M+H	PC(15:0/18:1(11Z))	2.82E-06	1.34E-11
1.57	802.5373	10.09	HMDB0008182	M+H	PC(18:3(6Z,9Z,12Z)/20:5(5Z,8Z,11Z,14Z,17Z))	4.31E-07	1.43E-11
1.12	702.5439	10.75	HMDB0008951	M+H	PE(16:0/P-18:1(11Z))	6.32E-04	1.72E-11
1.91	834.6049	10.67	HMDB0113305	M+H	PE-NMe(20:0/22:6(4Z,7Z,10Z,13Z,16Z,19Z))	5.77E-11	1.75E-11
1.79	482.3242	10.11	HMDB0010381	M+H	LysoPC(15:0/0:0)	2.40E-09	1.77E-11
1.44	764.5565	11.66	HMDB0008029	M+Na	PC(16:1(9Z)/P-18:1(11Z))	4.91E-06	2.04E-11
1.12	407.3017	8.26	HMDB0011543	M+Na	MG(0:0/20:1(11Z)/0:0)	6.14E-04	2.16E-11
1.08	159.0277	1.07	HMDB0000157	M+Na	Hypoxanthine	1.23E-03	2.69E-11
2.11	796.5260	11.09	HMDB0009710	M+Na	PE(22:6(4Z,7Z,10Z,13Z,16Z,19Z)/P-18:1(11Z))	2.37E-14	2.73E-11
1.16	757.5617	9.54	HMDB0114872	M+H	PA(16:1(9Z)/24:1(15Z))	3.48E-04	2.73E-11
1.53	820.5269	11.66	HMDB0112336	M+Na	PS(15:0/22:4(7Z,10Z,13Z,16Z))	1.00E-06	2.83E-11
1.95	188.0706	4.02	HMDB0000734	M+H	Indoleacrylic acid	1.69E-11	3.18E-11
2.04	424.3422	9.14	HMDB0006461	M+H	Linoelaidyl carnitine	4.76E-13	3.23E-11
1.72	548.3709	9.72	HMDB0010392	M+H	LysoPC(20:2(11Z,14Z)/0:0)	1.42E-08	5.02E-11
1.50	776.5732	10.24	HMDB0009644	M+H	PE(22:5(4Z,7Z,10Z,13Z,16Z)/P-18:1(11Z))	1.65E-06	5.25E-11

1.37	542.3240	8.51	HMDB0010397	M+H	LysoPC(20:5(5Z,8Z,11Z,14Z,17Z)/0:0)	1.61E-05	8.86E-11
1.31	468.3085	8.37	HMDB0010379	M+H	LysoPC(14:0/0:0)	4.79E-05	9.23E-11
1.59	550.3866	10.36	HMDB0010391	M+H	LysoPC(20:1(11Z)/0:0)	5.11E-07	1.11E-10
1.19	808.5815	10.38	HMDB0000593	M+Na	PC(18:1(9Z)/18:1(9Z))	3.29E-04	1.18E-10
1.29	269.0879	1.28	HMDB0000195	M+H	Inosine	1.03E-04	1.23E-10
1.34	111.0078	1.09	HMDB0000243	M+Na	Pyruvic acid	2.89E-05	1.28E-10
1.14	248.1493	1.16	HMDB0013127	M+H	3-Hydroxybutyrylcarnitine	4.85E-04	1.47E-10
1.99	144.0115	1.80	HMDB0003417	M+Na	D-Cysteine	3.13E-12	1.74E-10
1.10	537.3620	5.75	HMDB0115487	M+H	PA(8:0/16:0)	2.35E-03	1.90E-10
1.08	572.3713	9.59	HMDB0010401	M+H	LysoPC(22:4(7Z,10Z,13Z,16Z)/0:0)	1.00E-03	2.07E-10
1.29	733.5537	10.27	HMDB0114793	M+H	PA(14:0/24:0)	5.92E-05	2.10E-10
1.28	814.5718	11.09	HMDB0008457	M+Na	PC(20:4(5Z,8Z,11Z,14Z)/P-18:1(11Z))	7.26E-05	2.40E-10
1.61	506.3603	10.19	HMDB0010408	M+H	LysoPC(P-18:1(9Z)/0:0)	1.66E-07	2.53E-10
1.48	232.1544	3.60	HMDB0000736	M+H	Isobutyryl-L-carnitine	2.87E-06	2.55E-10
1.27	848.5942	11.10	HMDB0009113	M+Na	PE(18:2(9Z,12Z)/24:1(15Z))	8.51E-05	2.56E-10
1.16	391.3427	11.32	HMDB0240583	M+H	Arachidonoylcholine	3.87E-04	2.73E-10
1.26	590.3212	8.90	HMDB0010404	M+Na	LysoPC(22:6(4Z,7Z,10Z,13Z,16Z,19Z)/0:0)	1.06E-04	2.97E-10
1.53	621.3101	12.52	HMDB0061690	M+H	LysoPI(20:4(5Z,8Z,11Z,14Z)/0:0)	8.90E-07	4.49E-10
1.20	792.5886	11.05	HMDB0008095	M+Na	PC(18:1(11Z)/P-18:1(11Z))	2.19E-04	5.37E-10
1.22	527.4351	11.30	HMDB0071469	M+H	TG(8:0/8:0/i-12:0)	1.72E-04	5.44E-10
1.92	380.2562	8.29	HMDB0000277	M+H	Sphingosine 1-phosphate	4.06E-11	5.58E-10
1.16	882.5904	13.15	HMDB0008321	M+Na	PC(20:1(11Z)/22:6(4Z,7Z,10Z,13Z,16Z,19Z))	3.64E-04	7.06E-10
1.54	300.2896	11.65	HMDB0000252	M+H	Sphingosine	9.76E-07	7.92E-10
1.04	500.2748	8.88	HMDB0011477	M+Na	LysoPE(0:0/18:2(9Z,12Z))	2.00E-03	8.66E-10
1.56	532.3438	9.68	HMDB0011481	M+Na	LysoPE(0:0/20:0)	5.30E-07	8.80E-10
1.33	508.3395	9.05	HMDB0011482	M+H	LysoPE(0:0/20:1(11Z))	4.55E-05	1.01E-09
1.52	398.3265	8.96	HMDB0006317	M+H	trans-Hexadec-2-enoyl carnitine	1.23E-06	1.02E-09
1.96	438.2982	9.46	HMDB0011152	M+H	LysoPE(P-16:0/0:0)	1.10E-11	1.03E-09
1.28	833.6449	11.65	HMDB0042117	M+Na	TG(14:0/15:0/20:5(5Z,8Z,11Z,14Z,17Z))	7.15E-05	1.09E-09
2.06	163.1328	5.70	HMDB0033625	M+H	(3R,7R)-1,3,7-Octanetriol	4.21E-13	1.22E-09
1.02	530.3580	9.72	HMDB0013122	M+Na	LysoPC(P-18:0/0:0)	1.97E-03	1.25E-09
1.24	480.3037	5.67	HMDB0011475	M+H	LysoPE(0:0/18:1(11Z))	1.82E-04	1.34E-09
1.67	1109.6534	8.92	HMDB0116822	M+Na	CL(8:0/8:0/8:0/21:0)	4.88E-08	1.74E-09
1.18	396.3108	8.51	HMDB0013334	M+H	9,12-Hexadecadienoylcarnitine	3.24E-04	2.00E-09

1.14	872.6079	11.65	HMDB0009410	M+Na	PE(20:4(5Z,8Z,11Z,14Z)/24:1(15Z))	4.48E-04	2.01E-09
1.26	923.6897	5.26	HMDB0053013	M+Na	TG(18:3(6Z,9Z,12Z)/20:3(5Z,8Z,11Z)/18:3(6Z,9Z,12Z))	1.06E-04	2.10E-09
1.12	188.0686	2.30	HMDB0000159	M+Na	L-Phenylalanine	5.81E-04	2.15E-09
1.90	476.2749	9.18	HMDB0011473	M+Na	LysoPE(0:0/16:0)	1.21E-10	2.34E-09
1.67	837.6191	10.67	HMDB0115089	M+Na	PA(20:0/24:1(15Z))	4.79E-08	2.40E-09
1.26	689.4934	6.03	HMDB0007208	M+Na	DG(18:1(11Z)/22:6(4Z,7Z,10Z,13Z,16Z,19Z)/0:0)	4.09E-04	2.56E-09
1.02	262.1284	1.11	HMDB0061717	M+H	Succinylcarnitine	1.99E-03	2.75E-09
1.43	436.3057	8.60	HMDB0013333	M+Na	3-Hydroxy-9-hexadecenoylcarnitine	9.50E-06	2.89E-09
1.64	538.3877	10.74	HMDB0011490	M+H	LysoPE(0:0/22:0)	8.91E-08	3.17E-09
1.46	1091.5988	5.67	HMDB0116851	M+Na	CL(8:0/8:0/10:0/18:2(9Z,11Z))	6.92E-06	3.21E-09
1.11	902.4752	11.38	HMDB0012450	M+Na	PS(22:6(4Z,7Z,10Z,13Z,16Z,19Z)/22:6(4Z,7Z,10Z,13Z,16Z,19Z))	9.45E-04	3.38E-09
1.27	778.4470	0.77	HMDB0012339	M+Na	PS(14:0/20:4(5Z,8Z,11Z,14Z))	7.43E-05	3.52E-09
1.07	908.6505	10.16	HMDB0113770	M+H	PE-NMe(11D5/13M5)	1.09E-03	3.84E-09
1.68	536.3716	9.91	HMDB0011491	M+H	LysoPE(0:0/22:1(13Z))	5.70E-08	4.38E-09
1.70	127.0366	1.33	HMDB0000008	M+Na	2-Hydroxybutyric acid	2.24E-08	4.39E-09
1.30	718.4910	5.26	HMDB0009213	M+Na	PE(18:4(6Z,9Z,12Z,15Z)/P-16:0)	7.20E-05	4.65E-09
1.22	470.3206	5.41	HMDB0006455	M+Na	Arachidonoylcarnitine	4.17E-04	5.16E-09
1.04	1571.9675	9.24	HMDB0058282	M+Na	CL(18:1(11Z)/22:6(4Z,7Z,10Z,13Z,16Z,19Z)/18:1(11Z)/22:6(4Z,7Z,10Z,13Z,16Z,19Z))	2.30E-03	5.48E-09
1.59	422.3250	8.76	HMDB0000222	M+Na	L-Palmitoylcarnitine	2.54E-07	5.53E-09
1.03	522.2852	7.05	HMDB0000874	M+Na	Tauroursodeoxycholic acid	2.10E-03	5.87E-09
1.20	1005.7935	0.68	HMDB0050849	M+H	TG(20:1(11Z)/22:6(4Z,7Z,10Z,13Z,16Z,19Z)/22:6(4Z,7Z,10Z,13Z,16Z,19Z))	2.95E-04	9.80E-09
1.06	711.5243	5.35	HMDB0013462	M+Na	SM(d18:0/14:1(9Z)(OH))	1.78E-03	1.04E-08
1.35	556.3371	5.76	HMDB0011492	M+Na	LysoPE(0:0/22:2(13Z,16Z))	4.12E-05	1.05E-08
1.24	733.4881	5.31	HMDB0114827	M+Na	PA(15:0/22:4(7Z,10Z,13Z,16Z))	1.67E-04	1.24E-08
1.84	1001.6923	9.49	HMDB0061410	M+Na	PC(DiMe(13,5)/DiMe(13,5))	6.46E-10	1.54E-08
1.21	450.3575	9.38	HMDB0000848	M+Na	Stearoylcarnitine	1.87E-04	1.60E-08
1.91	382.2725	8.47	HMDB0001383	M+H	Sphinganine 1-phosphate	6.87E-11	1.63E-08
1.54	368.2398	5.03	HMDB0061637	M+Na	3-hydroxyundecanoyl carnitine	1.19E-06	1.95E-08
1.26	372.3110	10.72	HMDB0005066	M+H	Tetradecanoylcarnitine	9.10E-05	2.60E-08

1.07	190.0858	6.41	HMDB0002302	M+H	Indole-3-propionic acid	1.82E-03	3.85E-08
1.61	434.2856	5.21	HMDB0013335	M+Na	3-Hydroxyhexadecadienoylcarnitine	2.33E-07	3.94E-08
1.28	952.6765	10.26	HMDB0112735	M+Na	PS(22:0/24:1(15Z))	6.29E-05	4.42E-08
1.21	769.5552	6.56	HMDB0114831	M+Na	PA(15:0/24:0)	7.81E-04	4.84E-08
1.17	945.6708	5.26	HMDB0010536	M+Na	TG(20:5(5Z,8Z,11Z,14Z,17Z)/18:2(9Z,12Z)/20:5(5Z,8Z,11Z,14Z,17Z))	3.42E-04	6.10E-08
1.54	1019.7038	10.18	HMDB0010560	M+Na	TG(22:6(4Z,7Z,10Z,13Z,16Z,19Z)/20:5(5Z,8Z,11Z,14Z,17Z)/22:6(4Z,7Z,10Z,13Z,16Z,19Z))	7.42E-07	1.85E-07
1.02	632.4416	7.62	HMDB0008855	M+H	PE(14:1(9Z)/14:1(9Z))	2.41E-03	3.96E-07
1.04	535.0370	5.30	HMDB0001018	M-H	UDP-D-Xylose	5.51E-08	3.91E-04
1.33	757.4882	5.30	HMDB0010570	M+Cl	PG(16:0/16:0)	1.27E-07	4.65E-02
1.30	682.4041	5.41	HMDB0008856	M+Cl	PE(14:1(9Z)/15:0)	1.21E-13	6.19E-02
1.11	768.4937	5.49	HMDB0112331	M-H	PS(15:0/20:4(5Z,8Z,11Z,14Z))	3.45E-08	8.00E-02
1.60	470.3203	5.27	HMDB0006510	M-H	Cervonyl carnitine	1.89E-14	8.78E-02
1.17	732.4722	0.78	HMDB0009147	M+Cl	PE(18:3(6Z,9Z,12Z)/P-16:0)	1.15E-09	9.14E-02
1.05	841.5553	10.46	HMDB0115170	M+Cl	PA(20:4(5Z,8Z,11Z,14Z)/24:1(15Z))	2.87E-08	1.01E-01
1.03	800.6676	0.72	HMDB0011695	M-H	SM(d17:1/24:0)	2.92E-06	1.07E-01
1.67	756.4861	5.29	HMDB0112297	M-H	PS(14:0/20:3(5Z,8Z,11Z))	3.25E-13	1.38E-01
1.13	285.2064	8.82	HMDB0000672	M-H	Hexadecanedioic acid	5.77E-06	1.44E-01
1.14	674.5108	0.78	HMDB0008851	M-H	PE(14:0/P-18:0)	1.10E-09	1.45E-01
1.06	818.4440	0.77	HMDB0009210	M+Cl	PE(18:4(6Z,9Z,12Z,15Z)/22:6(4Z,7Z,10Z,13Z,16Z,19Z))	3.20E-07	2.24E-01
1.13	688.5242	0.78	HMDB0007895	M-H	PC(14:0/P-16:0)	1.06E-07	2.27E-01
1.18	751.4957	5.48	HMDB0114825	M+Cl	PA(15:0/22:1(13Z))	7.60E-09	2.37E-01
1.16	1502.0546	5.92	HMDB0057130	M-H	CL(18:0/18:1(11Z)/20:4(5Z,8Z,11Z,14Z)/20:4(5Z,8Z,11Z,14Z))	7.42E-09	2.44E-01
1.10	297.2428	9.52	HMDB0010736	M-H	3-Oxooctadecanoic acid	1.29E-07	2.90E-01
1.09	702.5282	0.79	HMDB0007961	M-H	PC(15:0/P-16:0)	8.12E-09	2.92E-01
1.20	781.4604	5.49	HMDB0114918	M+Cl	PA(18:1(11Z)/22:6(4Z,7Z,10Z,13Z,16Z,19Z))	3.12E-10	2.92E-01
1.22	990.7927	0.67	HMDB0008783	M+Cl	PC(24:0/24:1(15Z))	2.68E-11	2.97E-01
1.33	255.2325	11.53	HMDB0000220	M-H	Palmitic acid	8.90E-15	3.22E-01
1.44	277.2167	10.67	HMDB0001388	M-H	alpha-Linolenic acid	3.67E-16	3.22E-01
1.16	812.4044	0.78	HMDB0012351	M+Cl	PS(14:1(9Z)/22:6(4Z,7Z,10Z,13Z,16Z,19Z))	9.28E-10	3.31E-01
1.12	822.5689	5.42	HMDB0007887	M+Cl	PC(14:0/22:1(13Z))	5.17E-09	3.42E-01
1.34	279.2326	11.10	HMDB0000673	M-H	Linoleic acid	4.84E-15	3.44E-01
1.29	188.0170	1.62	HMDB0001476	M+Cl	3-Hydroxyanthranilic acid	2.55E-13	3.49E-01

1.14	734.4723	0.78	HMDB0008847	M-H	PE(14:0/22:6(4Z,7Z,10Z,13Z,16Z,19Z))	5.14E-09	3.50E-01
1.25	810.4076	0.78	HMDB0112496	M+Cl	PS(18:4(6Z,9Z,12Z,15Z)/18:4(6Z,9Z,12Z,15Z))	3.15E-12	3.53E-01
1.35	281.2481	11.66	HMDB0000207	M-H	Oleic acid	6.56E-15	3.60E-01
1.15	916.7056	0.69	HMDB0013449	M+Cl	PC(O-22:0/22:3(10Z,13Z,16Z))	1.76E-09	3.70E-01
1.18	101.0238	1.29	HMDB0000005	M-H	2-Ketobutyric acid	3.58E-08	3.84E-01
1.19	823.5543	5.63	HMDB0116585	M-H	PG(20:4(5Z,8Z,11Z,14Z)/20:1(11Z))	5.21E-10	4.04E-01
1.59	171.0654	5.38	HMDB0000341	M-H	2-Octenedioic acid	1.41E-20	4.04E-01
1.33	307.2635	11.80	HMDB0005060	M-H	Eicosadienoic acid	3.86E-11	4.24E-01
1.08	596.4013	5.18	HMDB0010699	M+Cl	CerP(d18:1/12:0)	8.99E-07	4.48E-01
1.07	750.5247	5.93	HMDB0007897	M+Cl	PC(14:0/P-18:1(11Z))	1.07E-07	4.49E-01
1.24	283.2638	12.53	HMDB0000827	M-H	Stearic acid	9.22E-12	4.49E-01
1.01	119.0342	0.76	HMDB0000337	M-H	(S)-3,4-Dihydroxybutyric acid	2.86E-07	4.50E-01
1.29	124.0096	0.78	HMDB0000251	M-H	Taurine	6.71E-11	4.76E-01
1.14	723.5022	5.48	HMDB0114846	M-H	PA(16:0/22:4(7Z,10Z,13Z,16Z))	3.08E-08	4.82E-01
1.08	720.5002	5.48	HMDB0009215	M-H	PE(18:4(6Z,9Z,12Z,15Z)/P-18:1(11Z))	1.88E-07	4.85E-01
1.16	530.3017	9.24	HMDB0010382	M+Cl	LysoPC(16:0/0:0)	1.03E-09	5.15E-01
1.06	154.0615	0.76	HMDB0000177	M-H	L-Histidine	1.01E-07	5.76E-01
1.12	303.2325	11.00	HMDB0001043	M-H	Arachidonic acid	1.91E-07	5.99E-01
1.05	89.0241	1.06	HMDB0000190	M-H	L-Lactic acid	2.68E-08	6.00E-01
1.05	327.2324	10.85	HMDB0002183	M-H	Docosahexaenoic acid	2.18E-06	6.25E-01
1.06	504.3092	8.92	HMDB0011483	M-H	LysoPE(0:0/20:2(11Z,14Z))	5.83E-05	7.00E-01
1.10	502.2932	9.22	HMDB0011484	M-H	LysoPE(0:0/20:3(11Z,14Z,17Z))	3.81E-04	2.35E+00
1.45	892.6483	6.32	HMDB0009245	M+Cl	PE(20:0/24:1(15Z))	1.75E-09	3.39E+00
1.67	652.4622	6.18	HMDB0010700	M+Cl	CerP(d18:1/16:0)	8.08E-15	4.91E+00

Figure S1. Microbial alpha diversity decreased in Unresectable PDAC and resectable PDAC patients shown by Chao 1 diversity index. (Chao1 index, $p = 0.81$).

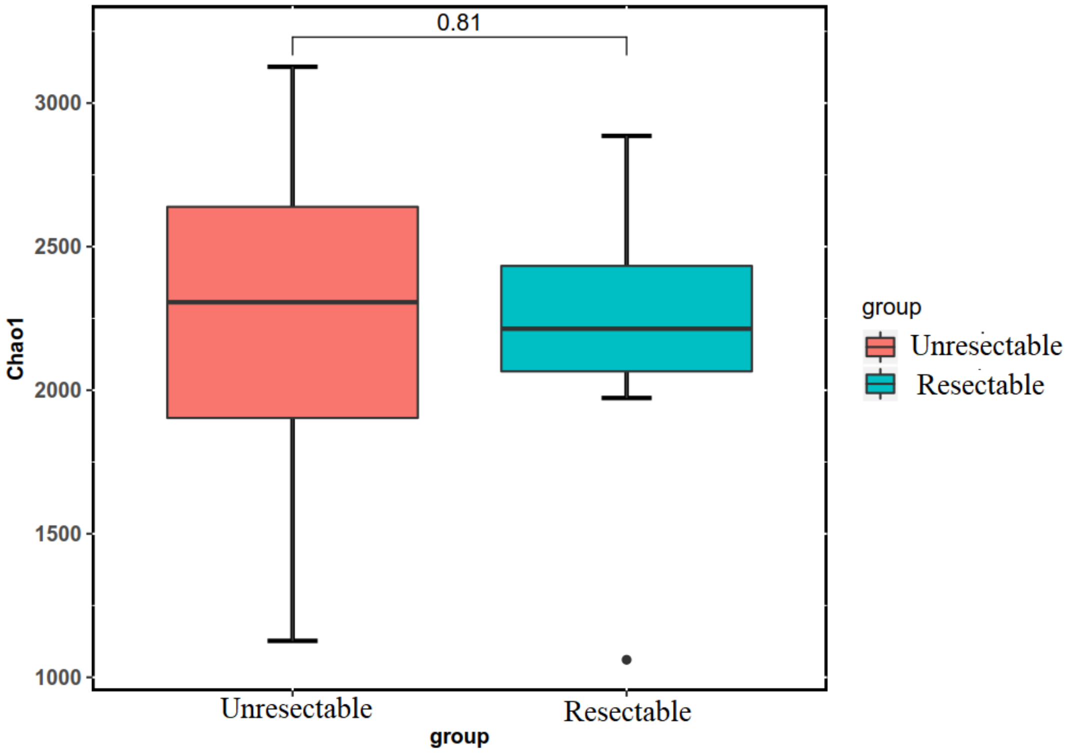

Figure S2. Relative abundance of the bacterial Species.

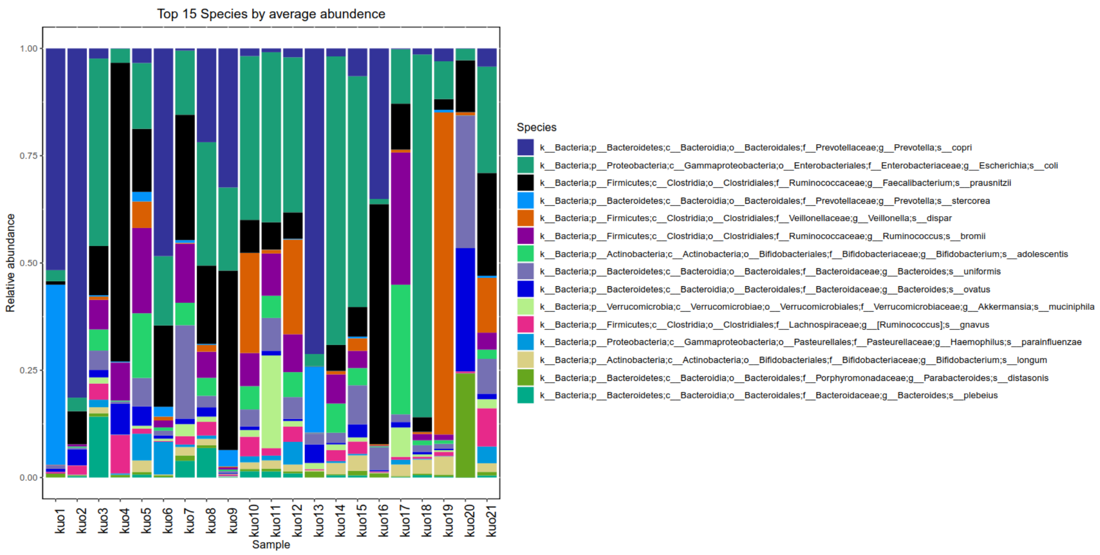

Supplement: Supplementary file 1 — Supplementary figures and table. [file jcav13p1555s1.pdf]
